# Supplementary material for: Trends in Early Sexual Initiation and Its Association with Socio-Environmental Factors among Korean Adolescents
Source: Children (Basel). 2023 Mar 24;10(4):613. doi: 10.3390/children10040613 (PMC10136636; doi:10.3390/children10040613)
Supplement: Supplementary file 1 [file children-10-00613-s001.zip › children-2265437-supplementary.pdf]

Supplementary Table S1. Socio-environmental characteristics of students younger than 13 years of age reporting sexual intercourse.

Variables	Subgroups	Number (Weighted Percentage)	
		2006–2008	2014–2016
**Total**		336 (100.0)	574 (100.0)
	**Family characteristics**		
Household income	Highest	75 (23.2)	135 (23.9)
	High	57 (16.3)	166 (29.4)
	Moderate	89 (24.2)	162 (28.1)
	Low	43 (14.3)	53 (8.3)
	Lowest	72 (22.0)	58 (10.3)
Residence type	With family	239 (68.2)	473 (82.0)
	With relatives	20 (5.3)	19 (3.1)
	With friends/alone [1]	22 (10.1)	28 (5.3)
	Care facility	55 (16.3)	54 (9.6)
Paternal education	≥College	101 (32.6)	232 (40.9)
	Completed high school	71 (18.0)	86 (14.3)
	≤Junior high school	22 (7.5)	22 (3.7)
	Unknown or deceased	142 (41.9)	234 (41.1)
Maternal education	≥College	77 (27.8)	222 (38.8)
	Completed high school	94 (25.3)	110 (18.1)
	≤Junior high school	23 (7.4)	19 (3.3)
	Unknown or deceased	142 (39.5)	223 (39.8)
	**School characteristics**		
Academic performance	Good–excellent	117 (33.3)	247 (43.7)
	Fair	79 (22.0)	139 (23.2)
	Poor–unsatisfactory	140 (44.7)	188 (33.1)
Composition	Coed	231 (73.6)	421 (75.6)
	Boys only	74 (18.4)	115 (18.6)
	Girls only	31 (8.0)	38 (5.8)
	**Community characteristics**		
Community type	Urban (Dong)	300 (95.0)	525 (92.9)
	Rural (Eup and Myeon)	36 (5.0)	49 (7.1)
City type	Large cities [3]	181 (56.3)	224 (38.2)
	Provincial areas [4]	155 (43.7)	350 (61.8)
Seoul Capital Area [5]	Yes	107 (46.1)	256 (50.5)
	No	229 (53.9)	318 (49.5)

The numbers of students reported specific age of their first sexual initiation in the 2006–2008 and 2014–2016 pooled data were 306 and 511, respectively. The mean ages (standard errors) of their first sexual debut were 11.46 (0.13) for the 2006–2008 pooled data and 10.93 (0.09) in 2014–2016. The mean ages were calculated excluding subjects who reported their age of sexual initiation before the age of 7 years old. [1] With friends/alone: living in a school dormitory or boarding house. [2] Specialized high school: High schools that focus on cultivating talent in specific fields, providing practice-oriented education designed for students who wish to get a job after graduation rather than apply for college. [3] Large cities: metropolitan cities (Seoul, Busan, Daegu, Incheon, Daejeon, Gwangju, and Ulsan) and the Special Self-Governing City, Sejong. [4] Provincial areas: provinces (Gangwon, Gyeonggi, Chungbuk, Chungnam, Jeonbuk, Jeonnam, Gyeongbuk, and Gyeongnam) and the Special Self-Governing Province, Jeju. [5] Seoul Capital Area: areas in and around the capital city (Seoul), including Incheon and Gyeonggi.
